# Systematic Review on the Impact of Intelligence on Cognitive Decline and Dementia Risk

**DOI:** 10.3389/fpsyt.2020.00658

**Published:** 2020-07-16

**Authors:** Francisca S. Rodriguez, Thomas Lachmann

**Affiliations:** ^1^ RG Psychosocial Epidemiology and Public Health, German Center for Neurodegenerative Diseases (DZNE), Greifswald, Germany; ^2^ Center for Cognitive Science, University of Kaiserslautern, Kaiserslautern, Germany; ^3^ Institute of Social Medicine, Occupational Health and Public Health (ISAP), University of Leipzig, Leipzig, Germany; ^4^ Facultad de Lenguas y Educación, Universidad Nebrija, Madrid, Spain

**Keywords:** intelligence, dementia, cognitive aging, Alzheimer disease, review

## Abstract

**Background:**

Previous studies have shown that an intellectually stimulating lifestyle is associated with a lower risk for cognitive decline and Alzheimer's disease and related dementia (ADRD). It is unclear so far whether higher intelligence may protect against this. The aim of this study was to conduct a systematic review on the association between intelligence and cognitive decline and ADRD risk.

**Methods:**

We searched the PubMed, web of science, and Scopus databases following the Preferred Reporting Items for Systematic reviews and Meta-Analyses (PRISMA) and Population, Intervention, Comparison, and Outcome (PICO) criteria. Quality of evidence was assessed using Critical Appraisal Skills Programme (CASP) checklists.

**Results:**

From an initial n=8,371 search hits, n= 14 studies met the inclusion criteria and had sufficient quality. Evidence indicates that cognitive decline in old age is not significantly associated with childhood intelligence (n=9). Evidence with regard to ADRD risk is inconclusive (n=5) with some studies showing no effects and other studies with significant effects having limitations in their design.

**Conclusions:**

Even though the majority of the studies show no significant association, we cannot exclude a possible effect that might be moderated by other, so far unknown factors. Further studies are necessary to systematically assess the influence of intelligence on ADRD risk and what factors moderate this association.

## Introduction

Alzheimer's disease and related dementias (ADRD) are one of the leading causes of morbidity and disability among older adults (1). It is estimated that today more than 35 million people live with ADRD worldwide and it is expected that this number will increase to 115 million in 2050 (2). ADRD is a chronic condition of the brain that is terminal and marked with deficits in memory, language, executive functions and other cognitive abilities so that the person's ability to manage daily living is severely impaired (3). Life expectancy for a person diagnosed with ADRD is between three to 12 years (4). Alzheimer's disease, the most common type of dementia (5), manifests through severe brain atrophy, amyloid beta accumulation in the brain, and hippocampal shrinkage (6). In any ADRD, there is a linear loss of brain volume and an acceleration of ventricular expansion (7). This seems to be the result of a loss of synaptic contacts in different brain areas (8) which manifests into a loss of intra- and internetwork connections over time (9). The exact processes of neurological degeneration in ADRD differ slightly between the different types of dementia, but altogether they are not well understood. It is clear, however, that these processes are connected to the aging of the brain (6).

Given the increasing prevalence of ADRD and the gross burden on the individuals and society that accompanies it, a lot of research effort is being made to identify risks and protective factors. Several studies show that an intellectually challenging lifestyle is associated with a slower cognitive decline in old age (10) and a reduced risk for ADRD (11, 12). However, there may be a reverse causality bias when interpreting these findings: the choice to have an intellectually challenging lifestyle may be related to intelligence, and intelligence might be the real factor protecting cognitive functioning against decline and ADRD.

The hypothesis that intelligence protects against age-related cognitive impairments can be derived from the fact that people with a higher intelligence demonstrate a better cognitive performance throughout the entire life-course (13). Evidence has shown the stability of IQ scores from ages 11 to 80 (14) and that brain volume accounts for about 15% of the variance in intelligence, in particular the grey matter in the frontal, temporal, parietal, and occipital lobes (15). Facing pathological brain atrophy, this greater brain volume might enable people of higher intelligence to have better cognitive functioning for a longer period in later life. Moreover, higher intelligence was found to correspond to a shorter path length in the brain network and a better global efficiency of the brain (16). Such efficient brain networks could also provide a form of protection in the event of damage. The *scaffolding theory of aging and cognition* assumes that some brains have the capacity to adapt to damage by functional reorganization (17). The capacity to efficiently use neural circuits to compensate for the impaired parts of the brain is explained by the *compensation-related utilization of neural circuits hypothesis* (CRUNCH). According to CRUNCH, fMRI activity increases when task difficulty increases because the person is compensating for the deficits in the neural network (18). The capacity to maintain a good cognitive functioning in face of ADRD pathology is referred to as *cognitive reserve* (19). People with high cognitive reserve seem to compensate for brain pathology by recruiting the contralateral dorsolateral prefrontal cortex (DLPFC) (20, 21). Further, people with high cognitive reserve tolerate more brain atrophy before engaging a compensatory network (22). However, even though cognitive reserve is considered the result of an intellectually challenging lifestyle (19), its relationship with intelligence is unknown.

In the present article, we provided a systematic review of the role of intelligence in withstanding ADRD. The review focuses on the association between intelligence in early and mid-life on cognitive decline and the risk for developing ADRD later in life. The hypothesis is that people with higher intelligence experience less cognitive decline and a lower ADRD risk than people with lower intelligence. We assume that this is due to the brain's ability to tolerate more damage and to compensate for damage by more efficient cognitive processing.

## Materials and Methods

### Inclusion and Exclusion Criteria

The criteria for inclusion and exclusion were determined by following the *Population, Intervention, Comparison, Outcome (PICO)* Model (23). For *population,* we included studies with samples from the general population and we excluded studies that focused on particular patient groups or clinical cohorts and studies that exclusively looked at specific subpopulations (e.g., patients with HIV or schizophrenia). For *intervention or prognostic factor,* we included studies on intelligence and mental ability in childhood and mid-life. We excluded studies that used proxies for intelligence (e.g., school grades). For *comparison,* we included longitudinal studies investigating cognitive decline and incident ADRD only. We excluded studies that assessed intelligence in later life as amyloid beta begins to accumulate up to 20 years before disease onset, which might have biased the estimation of intelligence (24). Case-control studies were excluded because it is unclear whether controls might have premorbid dementia symptomatology in the brain that has not yet been detected at the time of the study and/or develop dementia soon after the point of the study so that the conclusions drawn based on the comparison might be biased. For *outcome of interest,* we included studies on cognitive decline and on dementia in general or a specific type of dementia such as vascular dementia or Alzheimer's disease. We excluded studies that investigated only the level of cognitive functioning at one point of time because this did not provide information about resilience to age-related or pathological decline. Moreover, studies were also excluded when they were of poor quality due to their methodology.

### Search Strategy

The search strategy of this review followed the *Preferred Reporting Items for Systematic Reviews and Meta-analyses* (PRISMA). A literature search was conducted in the PubMed, Web of Science, and Scopus databases on June 4th, 2019. We did not filter the results in order to identify as many relevant studies as possible. The search string was composed of two parts: first, the search terms “intelligen* OR IQ” in the abstract or title, and second, the search terms “cognitive decline OR dementia OR Alzheime*” in the abstract or title. The Web of Science database did not permit the limitation of the search terms to abstract or title so instead, we performed a search by topic. In addition to the systematic literature search, a manual search was conducted using the references of the identified articles and related review articles.

### Data Extraction

The search results were imported directly from the database into the reference software Citavi. In the first step, four independent blinded reviewers screened the titles of the search results according to the inclusion and exclusion criteria. Additionally, we retained only those publications written in English that had an abstract and excluded all commentaries, editorials, conference abstracts, and book chapters. In the second step, two independent raters screened the abstracts of the remaining search results according to the inclusion and exclusion criteria and their ratings were discussed in group meetings. Based on the consensus of these group meetings, only studies that fulfilled the inclusion criteria remained in the evaluation process. In the third step, we screened the full-texts of the remaining search results. The full-text screening was conducted by two independent raters, who also evaluated the quality of the studies using the *Critical Appraisal Skills Programme* (CASP) checklist for cohort studies. The independent ratings by the two raters were compared and discussed until a consensus was reached. Studies with minor limitations and a minimum risk for bias (e.g., only few confounders, only elementary statistical models) were included in the results, if they met the inclusion criteria. Studies with major limitations or a moderate to high risk for bias (e.g., follow-up not long enough, validity of IQ assessment questionable) were excluded.

### Screening Process

**Figure 1:**
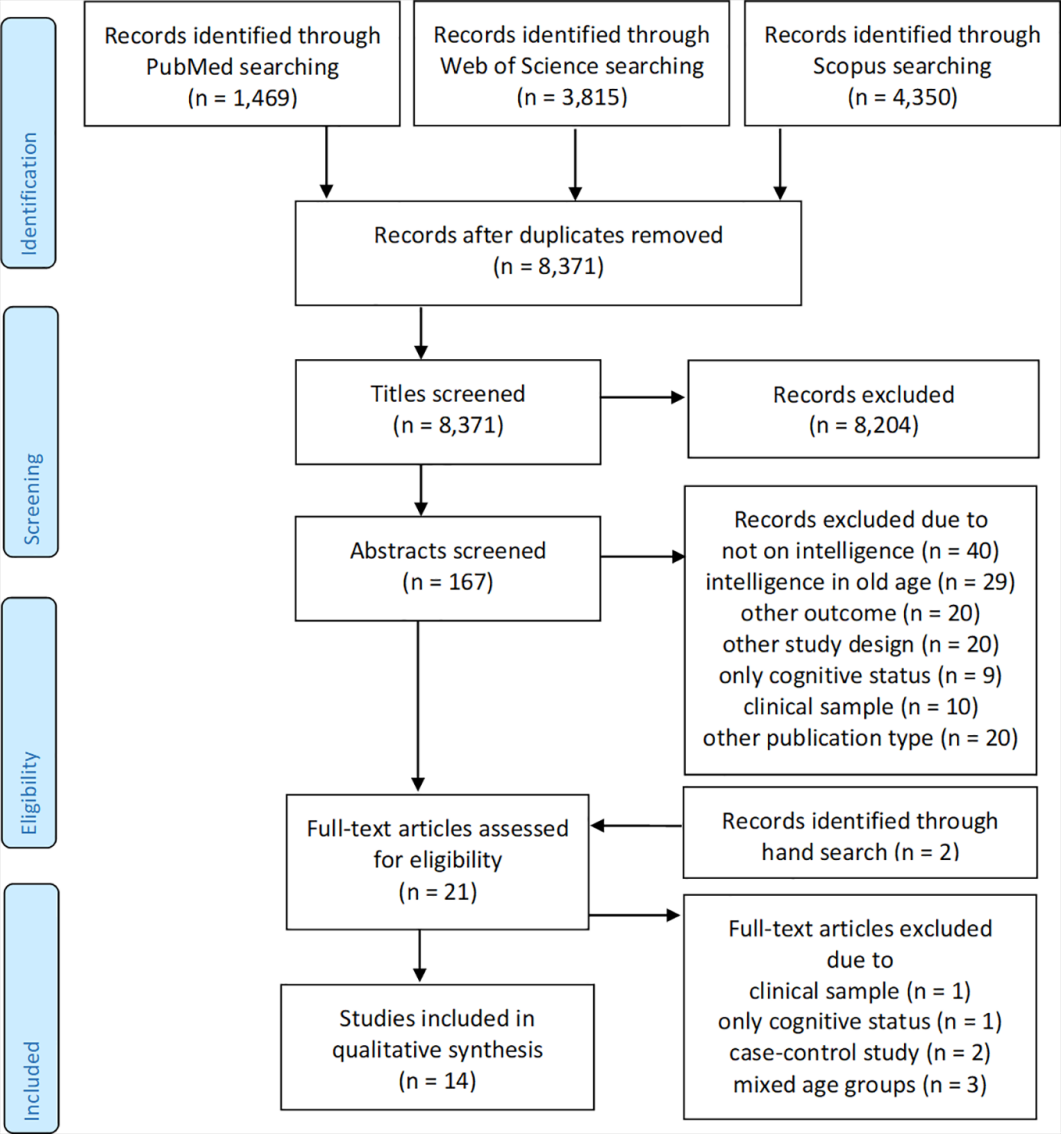
Flow diagram of the literature search process (according to prisma-statement.org).

The number of publications reviewed at each phase of the systematic review is graphically illustrated in **Figure 1**. The literature search in the databases revealed a total of n=1,469 search results in PubMed, n=3,815 in Web of Science, and n=4,350 in Scopus. After eliminating duplicates, a total of n=8,371 search hits remained for the title-screening. The title-screening was performed by four independent raters (one senior research scientist and three study assistants with a university degree in psychology or cognitive science). Rater 1 identified 1.13% of the studies as relevant, rater 2 identified 0.50% studies as relevant, rater 3 identified 0.29% of the studies as relevant studies and 0.48% as “maybe”-relevant, and rater 4 identified 0.68% of the studied as relevant and 1.62% as “maybe”-relevant. The general interrater reliability as estimated *via* Cohen's kappa was 0.181 (z=40.58, p < 0.001). Interrater reliability between the senior research scientist and the three raters were as follows: Rater 1 was 97.32% (Cohen's kappa 0.210, z=20.5, p < 0.001), rater 2 was 98.45% (Cohen's kappa 0.179, z=16.77, p > 0.001), and rater 3 was 98.84% (Cohen's kappa 0.287, z=28.51, p < 0.001). All titles with a rating of “yes” by any rater were included in the next step of the review, the abstract screening. All titles rated with “maybe” were reviewed according to the inclusion and exclusion criteria and evaluated in context with the ratings of the other raters. The titles that were considered relevant were included in the abstract screening. The title screening led to the elimination of many studies that did not meet the inclusion and exclusion criteria as well as the elimination of editorials, commentaries, book chapters, conference abstracts, and papers without abstracts. A total of n=167 relevant publications remained for the abstract screening.

The abstract screening was conducted by two independent reviewers (one senior research scientist and one study assistants with a university degree in psychology). Rater 1 included n=16 studies (9.6%) and rater 2 included n=30 studies (18.0%). The raters agreed in their ratings for n=149 studies (89.2%; Cohen's kappa 0.553, z=7.620, p < 0.001). The n=18 studies with disagreements were discussed in group meetings. As a result, n=13 of these studies were excluded because they did not meet the inclusion and exclusion criteria. In total, the abstract screening excluded n=9 publications because they investigated cognitive status instead of cognitive decline, n=40 because they did not focus on intelligence, n=20 because they had a different outcome of interest, n=20 because the study design and/or research question did not correspond to the research question for the review, n=29 because they assessed intelligence in old age, n=10 because the sample was comprised of patient groups, and n=20 because the publication type was not relevant (e.g., conference abstracts, editorials, commentaries, without abstract, foreign language). A total of n=19 articles met the inclusion and exclusion criteria and underwent the full-text screening. A search of the reference lists of these articles revealed an additional n=2 relevant studies that were also included in the full-text screening.

The full-text and quality screening of the n=21 articles led to the exclusion of n=7 studies. One study was excluded because the authors used a sample of Alzheimer patients and studied them retrospectively without a control group (25). Another study was excluded because there was only one time point with a cognitive measure in old age, so there was no indication about how cognition changed in old age (26). Two studies were excluded because they were case-control studies: one study linked hospital records to participants from the Scottish Mental Health Survey (27) and another study linked hospital and mental health records to LBS participants (28). Three studies were excluded because they modeled cognitive change over time, which did not allow for the interpretation of the association between the IQ and cognitive decline in old age (29–31). The overall quality of the remaining studies was judged to be good or very good and thus no study was excluded due to poor quality. A total of n=14 studies met the inclusion and exclusion criteria as well as the quality standards. A summary of the studies is shown in **Table 1**.

**Table 1:** Results of the systematic literature search.

Authors	Population	IQ assessment	Outcome	FU time	Effect size	Comments
Cognitive Decline						
Gow et al. (32)	Lothian Birth Cohort^§^ of 1921, Scotland, n=543	Moray House Test, age 11	Decline of performance in Raven's Matrices, Verbal Fluency, and Logical Memory, age 79-83	68-72 years	Latent cognitive functioning variable on decline of performance in any test: non-significant (latent variable growth curve modeling adj. sex, education, occupational social class, smoking, alcohol intake)	short observation period of only 4 years
Ritchie et al. (33)	Lothian Birth Cohort^§^ of 1921, Scotland, n=1091	Moray House Test, age 11	Decline of performance in subtests of Wechsler Adult Intelligence Scale, age 69-76	58-65 years	Moray House Test on decline of performance in any test: non-significant (latent growth curve model adj. sex, education, parental occupational status, own occupational status, deprivation of the residential area, physical fitness, APOE e4 status, smoking, body mass index, cardiovascular disease, hypertension, and diabetes)	
Salarirad et al. (34)	Lothian Birth Cohort^§^ of 1921, Scotland, n=106	Moray House Test, age 11	Decline of performance in Raven's Progressive Matrices, age 77-80	66-69 years	Moray House Test on decline of performance in the Raven's Progressive Matrices: non-significant (latent growth modeling adj. sex, age, brain white matter hyperintensities)	only one cognitive domain as outcome, adjusted only a few confounders, very small number of study participants
Staff et al. (35)	Lothian Birth Cohort^§^ of 1936, Scotland, n=388	Moray House Test, age 11	Decline of performance in Rey Auditory-Verbal Learning Test, age 64-77	53-66 years	Moray House Test on decline of performance in the Rey Auditory-Verbal Learning Test: non-significant (multilevel linear modeling adj. age, sex, practice, education, SES in childhood, mobility)	statistical reporting is not very comprehensive, only verbal learning test as outcome
Staff et al. (36)	Lothian Birth Cohort^§^ of 1921 and 1936, Scotland, n=751	Moray House Test, age 11	Decline of performance in Raven's Progressive Matrices, age 62-83	51-72 years	Moray House Test on decline in performance in the Raven's Progressive Matrices: non-significant (linear mixed modeling adj. for age and practice)	adjusted for only two confounders, research question of the paper is different than the research question of the review
Bourne et al. (37)	Lothian Birth Cohort^§^ of 1921 and 1936, Scotland, n=91; n=349	Moray House Test, age 11	Decline of performance in the Raven's Progressive Matrices, age 77-80 and age 64-66, mean interval 2 years	53-69 years	Moray House Test on decline of performance in the Raven's Progressive Matrices: b = 0.13, p < 0.001 (multiple linear regression adj. gender, education, occupational status, smoker, alcohol, cohort, interval between testing)	only one cognitive domain as outcome, very small number of study participants, short observation period of only 2 years on average
Gow et al. (38)	Lothian Birth Cohort^§^ of 1921 and 1936, Scotland, n=496+1028	Moray House Test, age 11	Decline of performance in Moray House Test, age 79-86	68-75 years	Moray House Test on decline of performance in Moray House Test: non-significant (growth curve model adj. age, sex, social class, number of years of education, smoking status, alcohol consumption)	included only dementia-free individuals
Gow et al. (39)	Lothian Birth Cohort^§^ of 1921 (LBC), Scotland, n=548; National Survey of Health and Development (NSHD), Great Britain, n=3262	Moray House Test, age 11; NSHD-devised test of verbal and non-verbal ability, age 15	Decline of performance in Raven's Progressive Matrices, Verbal Fluency, Logical Memory, age 79, 83, 87; Verbal Memory, Search Speed, age 43, 53	32-76 years	Moray House Test on decline of performance in any test: non-significant (latent growth curve modeling adj. sex, social class, education, smoking, alcohol consumption)	adjusted only for a few confounders, model shape does not replicate the natural cognitive decline
Richards et al. (40)	National survey of health and development, Great Britain, n=2058	Heim AH4 test, Watts-Vernon reading test, age 15	Decline of performance in word list learning, visual search, age 43-53	28-38 years	Cognitive ability score on decline of performance in memory: men b=0.09, p = 0.005, women b=0.10, p < 0.001; Search speed: men b=0.13, p < 0.001, women b=0.08, p=0.01 (conditional models adj. memory and speed scores at 43 years, educational attainment, occupational social class, health indicators)	only linear analysis
***Alzheimer's Disease and Related Dementias (ADRD)***						
Fritsch et al. (41)	Cleveland Longitudinal Aging Studies of Students (CLASS), OH, USA, n=396	Otis Self-Administering Test of Mental Ability, age 15	Dementia accord. DSM-IV and MCI (Modified telephone interview for Cognitive Status, proxy respondents), mean FU age 75	about 60 years	Mental ability score on dementia: OR 0.55, CI 0.30–1.00; MCI: OR 0.46, CI 0.24–0.84 (logistic regression analysis adj. for sex, activity level, and education)	adjusted for only a few confounders, not enough socioeconomic variance because of affluent neighbourhood, only one FU point for following up on dementia prevalence
Huang et al. (42)	Project Talent–Medicare (PT-MED), USA n=85,763	Battery of adolescent cognitive aptitude tests, age 14-18	Dementia (Medicare-recorded codes), age 68-74	53-56 years	Cognitive ability score on dementia: men: OR 1.17, CI 1.04-1.32, women: OR 1.17; CI 1.04-1.31; Memory for words on dementia: men OR 1.16, CI 1.05-1.27, women OR 1.16, CI 1.05-1.28; Word function in sentences on dementia: men: OR 1.13, CI 1.03-1.25, women: OR 1.14, CI 1.03-1.27; Reading comprehension on dementia: men: OR 1.15, CI 1.02-1.29, women: OR 1.14, CI 1.02-1.27; Abstract reasoning on dementia: men: OR 1.12, CI 1.01-1.24, women: OR 1.13, CI 1.01-1.25; Introductory math on dementia: men: OR 1.15, CI 1.03-1.28, women: OR 1.11, CI 1.01-1.22; Arithmetic computation on dementia: men: OR 1.15, CI 1.04-1.27, women: OR 1.14, CI 1.03-1.26 (logistic regression stratified by sex, adj. birth year, race, adolescent SES, region of school, region of residence)	only Medicare participants, no validation of dementia diagnosis, advantage of the study is that it is a nationally representative study of American high school students
Osler et al. (43)	Danish Conscription Database, Denmark, n=666,986 men	Børge Prien Prøve draft board intelligence test, age 20	Dementia (psychiatric/somatic hospital/registers, age 10-77	Up to 57 years	Børge Prien Prøve draft board intelligence test on dementia: HR 1.74, CI 1.60–1.88; Vascular dementia HR 1.47, CI 1.31–1.56; Alzheimer's disease HR 1.07, CI 1.03-1.13; Twin brothers: HR 1.36, CI 1.08–1.73 (cox regression with age as time scale, Fine-Gray competing risk, adj. for height, education, age)	included only men eligible for military service, no validation of dementia diagnosis, adjusted only few confounders
Rantalainen et al. (44)	Helsinki Birth Cohort Study, Finland, n=2,785 men	Finnish Defence Forces Basic Intellectual Ability Test, age 20	Dementia (hospital discharge/death registers, outpatient records), age 25-79	Up to 59 years	Cognitive ability total score on dementia: HR 1.22, CI 0.97-1.54; Verbal ability on dementia: HR 1.14, CI 0.90-1.43, Arithmetic on dementia: HR 1.22, CI 0.96-1.53, Visuospatial abilities on dementia: HR 1.27, 1.00-1.60 (cox proportional hazard models adj. birthweight, mother's age, father's occupational status in childhood, attained level of education in adulthood, stroke, coronary heart disease)	included only men eligible for military, no validation of dementia diagnosis
Russ et al. (45)	Lothian Birth Cohort^§^ of 1921, Scotland, n=32,467	Moray House Test, age 11	Dementia (psychiatric/hospital/death records, subset primary care records), age 60-96	49-85 years	Moray House Test on dementia: women: HR 1.51, CI 1.29-1.76, men: HR 1.19, CI 0.98-1.44; women with IQ 100-114.9: HR 1.18, CI 1.03-1.34; women with IQ 85-99.9: HR 1.32, CI 1.15-1.51; men with IQ non-significant (hierarchical cox proportional hazards model with school- and county-level random effects adj. socioeconomic position, inverse probability weighting for survival)	adjusted for only a few confounders, no validation of dementia diagnosis, advantage of the study is that it adjusted for loss to follow-up by using weights and intensive record screening

§, the Lothian Birth Cohorts of 1921 and 1936 are assessed at the age of 11, an assessment named the Scottish Mental Survey; adj., adjusted; CI, 95% confidence interval; OH, Ohio; OR, odds ratio; USA, United States of America; LBC, Lothian Birth Cohort; NSHD, National Survey of Health and Development.

## Results

### Study Quality

A majority of the identified studies analyzed the *Lothian Birth Cohort* (LBS), which assesses childhood intelligence at the age of 11. Other studies used data from the *British National Survey of Health and Development* (NSHD), which assesses intelligence at the age of 15, from high school intelligence tests, or from cognitive aptitude tests completed before mandatory military training at age of 20. The follow-up periods varied between 28 to 96 years with some studies assessing cognitive decline between age 43 and 53 and others tracking health records either till death or the latest day of the study. However, most of the studies ended at or before the age of 80, so it is not possible to draw conclusions on the impact of intelligence on cognitive health in older ages. Another caveat is that studies using medical records did not validate the diagnostic status of the patient. Thus, it is unclear to what extent the fact that people who do not access or use medical services regularly were not included in the sample might have biased the results. With respect to quality, it is important to note that many studies adjusted only for a small number of confounders (e.g., sex, age, and white matter lesions (36). It is known that health status, especially cardiovascular health and depression, can affect cognitive functioning and dementia risk but most of the studies did not take that into account. Moreover, the European studies, as well as studies including veterans, did not account for problems due to war. Experiences of violence, trauma, deprivation, and shattered family relations would certainly affect people, but it is unclear to what extent they also affect the association between intelligence and ADRD.

Another quality issue that must be considered is that most of these studies are based on the LBS, which suffered a great loss to follow-up. Consequently, those most likely to be sick or those lost due to early death are not included in the analysis. It is likely that the effects reported from these studies have a healthy survivor bias, as non-responders might have had poorer cognitive health. Therefore, these studies could have a Type II error. In order to derive a true estimation of the effect of intelligence on cognitive health in later life, a cohort study that starts in midlife and continuously follows up on the participants over a minimum period of 30 years is necessary.

The following section presents the findings narratively, first, for cognitive decline and then for dementia. A narrative synthesis of the results was selected because of differences in study design and statistical methods used in the studies and because the majority of studies were derived from the same sample.

### Cognitive Decline

Associations between childhood intelligence and cognitive decline in old age were investigated by two studies using the NSHD and by eight studies using the LBS (adding to a total of n=9 publications because one study used both, the NSHD and LBS, cohorts). Details are shown in **Table 1**. The NSHD was conducted by the Medical Research Council, initially as a maternity survey of all births recorded in England, Scotland and Wales during 1 week of March, 1946. Individuals have been followed up since then. The LBS is a study that tested the intelligence of almost every child born in 1921 or 1936 at the age of 11 that attended school in Scotland. The follow-up studies of the LBS are also referred to as the Scottish Mental Surveys of 1932 and 1947.

In the first study, Richards and colleagues (2004) used data from the NSHD that assessed childhood intelligence *via* the Heim AH4 and the Vernon reading test at the age of 15 (n=2,058) (40). Linear regression analysis of the association between childhood intelligence and cognitive decline in word list learning and visual search at the ages 43 to 53 indicated that those with higher intelligence in childhood had a slower cognitive decline in their 40s and 50s [occupation not significant (n.s.), education significant]. A limitation of the study is that the authors did not use fixed-effects or growth curve models. Another study used NSHD data and analyzed it together with data from the LBS (n=548+3262) (39). Using latent growth curve modeling, childhood intelligence was associated with cognitive status at the age of 43 but not with cognitive decline between ages 43 to 53. Results for the LBS were similar: childhood intelligence as assessed *via* the Moray House Test at the age of 11 was not associated with the slope of cognitive decline (in Raven's Progressive Matrices, Verbal Fluency, Logical Memory) from the age of 79 to 87 [education n.s. (39)].

Data from the LBS was used in another seven publications, of which three investigated the decline in performance in the Raven Progressive Matrices (RPM) and four investigated performance in other cognitive tests (for details see **Table 1**). A study by Bourne and colleagues (2007) analyzed the association between childhood intelligence (Moray House Test) at the age of 11 and decline in RPM performance from age77 to 80 for those born in 1921 and age 64 to 66 for those born in 1936 (n=91+349) (37). Analysis *via* linear regression analysis indicated that people with a higher childhood intelligence had a slower cognitive decline (occupation n.s., education significant). A study by Salarirad and colleagues (2011) analyzed only the decline from age 77 to 80 and found no significant effect using latent growth modeling (n=106) (34). A study by Staff and colleagues (2014) also used LBS data to analyze the association between childhood intelligence and decline in RPM from age 62 to 83 (n=751) (36). Using linear mixed modeling, they found no significant associations. A study by Gow and colleagues (2008) that also used LBS data came to similar findings (n=543) (32): Decline of performance in the RPM as well as in Verbal Fluency and Memory from the age of 79 to 83 was not significantly associated with childhood intelligence using latent growth curve modeling (occupation n.s., education n.s.; despite the association being significant with linear regression modeling). Three other studies analyzed the impact of childhood intelligence with LBS data and found non-significant effects regarding decline of performance in the Rey Auditory-Verbal Learning Test from age 64 to 77 (n=388, education significant) (35), in the Moray House Test from the age 79 to 86 (n=496+1028, education n.s.) (38), and in the subtests of the Wechsler Adult Intelligence Scale from the age 69 to 76 (n=1,091, education n.s.) (33).

In summary, although a few studies that used linear regression analysis demonstrated associations between childhood intelligence and cognitive decline in old age, the majority of the studies cannot confirm such an association. Notably, if studies repeated the analyses that were initially conducted *via* linear regression analysis with growth curve models, the results became non-significant. It is important to note that most of the evidence comes from the LBS study and incurred substantial loss to follow-up.

### Alzheimer's Disease and Related Dementia (ADRD)

Associations between childhood intelligence and incident ADRD were investigated by five studies. Russ and colleagues (2017) made the effort to go through psychiatric, hospital, death, and primary care records of almost all LBS participants born in 1921 (n=32,467) to identify those who developed ADRD from ages 60 to 96 (45). Using hierarchical cox proportional hazards models with random effects for school and county as well as inverse probability weighting for survival, their results indicate that a lower childhood intelligence was associated with a higher risk for ADRD in women (HR 1.51), but not in men (HR 1.19; details in **Table 1**).

An even larger study (n=85,763) used a nationally representative sample of US-American high school students who took cognitive aptitude tests at the age of 14 to 18 and identified those who developed ADRD *via* Medicare records (a nationwide US health insurance program for people aged 65 and older) (42). The results indicate that lower intelligence in the teenage years was associated with a higher risk to develop ADRD (men OR 1.17, women OR 1.17). All cognitive abilities in teenage years were significantly associated with ADRD risk up to the age of 74 (see **Table 1** for details). Another study also used data from cognitive aptitude testing in high school in their analysis: Fritsch and colleagues (2005) invited high school students from Cleveland, Ohio, who had completed a cognitive aptitude test, to a cognitive screening at the age of 75 (n=396) (41). Their results also suggested that higher intelligence protects against ADRD (OR 0.55) and mild cognitive impairment in old age (OR 0.46; education n.s.).

Furthermore, two studies used the intelligence ability testing that men completed before mandatory military training at an average age of 20. Osler and colleagues (2017) used data from the Danish Conscription database and investigated how many men developed ADRD before the age of 77 (according to hospital registers, n=666,986) (43). They found significant associations between lower intelligence at the age of 20 and a higher ADRD risk (OR 1.74), even after taking loss to follow-up into account. Analysis with twin pairs indicated that the twin brother with lower intelligence had a higher risk for developing ADRD (HR 1.36) than the twin brother with higher intelligence (43). Education was significant but had only a small effect on the association. Rantalainen and colleagues (2018) used data from the Helsinki Birth Cohort Study and analyzed the association between Defense Forces Basic Intellectual Ability Test at the age of 20 and dementia before the age of 79 (according to hospital and outpatient records, n=2,785) (44). They did not find a significant association between intelligence and ADRD incidence (HR 1.22, education attenuated the effect); only poorer visuospatial abilities predicted ADRD incidence (HR 1.27). In contrast to Osler et al., the study team adjusted the analyses for birthweight, cardiovascular health, mother's age, and father's occupational status in childhood.

In summary, the largest medical record screening of the LBS, with a considerable age range (60-92 years), came to the conclusion that lower childhood intelligence is only associated with an ADRD risk in women. However, results from the largest US-representative study observed a significant association in both genders. It is important to note that neither of these studies adjusted for education. Furthermore, studies that used the military conscription data observed a moderate effect of education, with only one study concluding that intelligence had an independent effect on ADRD risk (43).

## Discussion

The aim of the present study was to review evidence on the association between intelligence in early and mid-life on cognitive decline and the risk for developing ADRD later in life. A systematic literature review identified nine studies on cognitive decline and five studies on dementia risk. Studies on cognitive decline suggest that there is no significant association with childhood intelligence. Importantly, most of those studies used the data from the Lothian Birth Cohort of 1921, all of them showing non-significant effects (32–34, 36, 38) with the exception of one study (37) that used only linear regression for analysis instead of more suitable models for longitudinal data. Studies on ADRD tend to show significant effects, but the lack of adjustment for numerous confounders coupled with the non-significant finding from a strong study call into question the validity of the these significant effects. Although a large U.S.-representative study observed a significant association between lower childhood intelligence and increased ADRD risk, a large-scale follow-up of the LBS observed this association only among women. Both studies were not adjusted for education, which is known to be a major factor influencing the risk for developing ADRD (46). As these studies assessed intelligence at the age of 11 to 18, subsequent education might have influenced ADRD risk. With regard to the two studies with men that were adjusted for education (both assessed intelligence at the age of around 20 years), one reported significant effects while the other did not. The study with no significant effects is the only study that adjusted for birthweight and cardiovascular health. As these confounders are well-known risk factors for ADRD (47–49), it is possible that their omission has biased the results. It is also worth noting that we excluded two studies that used school grades as proxies for intelligence that both have observed a significantly lower ADRD risk for people with better school grades (50, 51). However, school grades also represent motivation and motivation itself seems to affect ADRD risk (52, 53).

There is an interesting finding in one of the identified studies: among twin brothers, the twin with lower intelligence had a higher ADRD risk (43). By taking into account genetic predispositions for intelligence among the twins, this finding indicates that lifestyle factors, which influence intelligence into a person's early 20s, might determine ADRD risk. Previous studies suggest that such lifestyle factors include the mother's behavior, her developmental beliefs, mental health and level of education, family social support, family size, a disadvantaged social status (54), or exposure to high levels toxins like fluoride, arsenic, and lead (55, 56). ADRD researchers already suspect that the risk for developing ADRD is determined by multiple risk factors (57). Accordingly, lifestyle factors affecting intelligence in childhood are simply an addition to the pool of risk factors.

Results are diverging with respect to cognitive decline and ADRD. While there seems to be no significant effect of higher intelligence on cognitive decline, there might be an association with lower dementia risk. One explanation for this finding is that more intelligent people start at a higher cognitive ability level and decline at the same speed as other people. They would then reach the threshold for dementia diagnosis later compared to people with lower intelligence. If this is the case, then intelligence does not protect against developing ADRD in general but simply delays the point of onset. Research has shown that the brain networks of people with higher intelligence have shorter path lengths and thus better cognitive ability (58). With aging, this white matter consistently decreases even when taking into account childhood intelligence (59). In ADRD, the synaptic contacts in the brain deteriorate (60) leading to a loss of connections in the brain network (61), which suggest that people with higher intelligence might be similarly affected by dementia as people with lower intelligence. It is possible that intelligence mitigates ADRD risk simply by enhancing brain efficiency throughout the disease process, for instance by functional reorganization [e.g., *scaffolding theory* (17)] or the efficient use of neural circuits [e.g., *CRUNCH* hypothesis (18)]. However, more studies are required to validate these assumptions.

There are some limitations that must be mentioned. First, we can only summarize findings that have been published. It is possible that, due to a publication bias, non-significant findings were not published. In this case, assuming an association between intelligence in early life and ADRD risk/cognitive decline would be an overestimation of a non-existent effect. Second, we could identify only electronically published articles in English that are available in one of the scientific databases. It is not clear whether there are articles published in a foreign language or articles that were not published digitally report important findings that we have missed. Third, our results depend on the search terms used and an article using alternative words to describe the same effect might not have been discovered. Finally, our conclusions depend on the quality of the studies included in the review. If more studies with a higher level of quality were conducted, the conclusions might be different.

## Conclusion

The evidence underlying this review indicates that there seems to be little to no association between early- and mid-life intelligence and cognitive decline and that there seems to be a tendency for a significant association for developing ADRD in later life. This is, however, not supported by all studies. Further studies might help to provide clarification by systematically assessing the influence of factors that shape intelligence and how those, together with genetically determined intelligence, shape cognitive decline and/or ADRD risk. Overall, it seems that other lifestyle and environmental factors play a greater role in determining cognitive health in old age than intelligence. Nonetheless, intelligence might play a role in so far that it can determine the effects of exposure to certain types of environmental risk factors or protectors – an aspect that has not yet received much attention in clinical health research. From a clinical perspective, it is important not to underestimate the impact of lifestyle factors. Further, it is important to be aware that people with higher intelligence are not exempt from developing dementia and are similarly affected by cognitive decline. However, initially better cognitive skills might mask cognitive deterioration for a longer period of time, so clinicians and general practitioners should also focus on declining cognitive abilities and not just obvious cognitive impairments.

## Data Availability Statement

The original contributions presented in the study are included in the article/supplementary material, further inquiries can be directed to the corresponding author.

## Author Contributions

FR: Conceptualization, Literature review, Formal analysis of identified articles, Methodology, Project administration, Supervision of study assistants, Writing—original draft. TL: Conceptualization, Formal analysis of identified articles, Supervision of the study, Validation, Writing—review and editing.

## Funding

This work was supported by the Hans and Ilse Breuer Foundation. The sponsor had no role in the design, methods, review process, interpretation, and preparation of paper.

## Conflict of Interest

The authors declare that the research was conducted in the absence of any commercial or financial relationships that could be construed as a potential conflict of interest.
